# Hormone Therapy: A Potential Risk Factor Affecting Survival and Functional Restoration of Transplanted Lymph Nodes

**DOI:** 10.3389/fphar.2022.853859

**Published:** 2022-03-31

**Authors:** Dong Dong, Heng Wang, Liang Chen, Wei Wang, Tianyi Liu

**Affiliations:** ^1^ Department of Plastic and Aesthetic Surgery, Huadong Hospital Affiliated to Fudan University, Shanghai, China; ^2^ Shanghai Medical College of Fudan University, Shanghai, China

**Keywords:** lymph node transplantation, estrogen, tamoxifen, lymphatic system, hormone therapy

## Abstract

**Background:** Transplantation of lymph nodes (LNs) is an increasingly popular option for treating lymphedema. Increasing evidence indicates an intrinsic correlation between estrogen signaling and the lymphatic system. We explored the effects of 17β estradiol and antiestrogen treatment (tamoxifen) on the survival and functional restoration of transplanted popliteal lymph nodes (PLNs).

**Methods:** A total of forty-eight ovariectomized mice were divided into three groups of 16: OVX + E2 (treated with 17β-estradiol), OVX + TMX (treated with tamoxifen), and OVX (control; treated with olive oil as a solvent). After 2 weeks, PLNs were transplanted. Then, reconnections of lymphatic vessels were observed, and the morphology and survival of transplanted PLNs were evaluated 4 weeks after transplantation. T cells, B cells, lymphatic vessels, and high endothelial venules (HEVs) were subjected to immunofluorescence staining or immunohistochemical staining and quantified.

**Results:** The percentage of lymphatic reconnections was 93.75% in the OVX + E2 group, 68.75% in the OVX + TMX group, and 75% in the OVX group. Surviving PLNs were observed in 16 of 16 in the OVX + E2 group, seven of 16 in the OVX + TMX group, and 13 of 16 in the OVX group. The mean size of PLNs in the largest cross section of the OVX + TMX group was significantly lower than that in the other groups. The distributions of B cells and T cells in surviving PLNs were similar to those in normal LNs. The ratio of dilated HEVs/total HEVs and density of lymphatic vessels in the OVX + E2 group were the highest among the three groups, whereas the lowest ratio and density were observed in the OVX + TMX group.

**Conclusion:** Tamoxifen treatment might lead to cellular loss of transplanted LNs and interfere with the structural reconstruction and functional restoration, thereby inhibiting the survival of transplanted PLNs. Estrogen treatment facilitated the maintenance and regeneration of functional HEVs as well as lymphangiogenesis.

## Introduction

The lymphatic system (LS) is a unidirectional transport network which drains interstitial fluid into the blood circulation. The LS has a pivotal role in regulating body-fluid homeostasis, immune surveillance, and absorption of dietary fat (González-Loyola and Petrova, 2021). LS dysfunction can damage normal lymphatic backflow and lead to lymphedema. The latter is characterized by fluid accumulation, local adipose hyperplasia, and tissue fibrosis, which can impair the quality of life of patients dramatically (Iyer et al., 2020). Numerous factors are responsible for LS damage, including trauma, infection, and iatrogenic injury. Surgery or radiotherapy of tumors is the leading cause of lymphedema, especially in breast cancer and melanoma (Kataru et al., 2019). Despite recent improvement in surgical methods, LS damage remains a frequent complication of cancer surgery (Rockson et al., 2019). Increasing evidence (Fish et al., 2020; Byun et al., 2021) indicates that >20% of patients suffer from lymphedema after breast cancer treatment; lymphedema has become a non-negligible complication of breast cancer therapy.

Estrogen, especially 17β-estradiol (E2), is a steroid hormone with very complex biological functions, which are involved in reproduction, neuroendocrine, vascular, skeletal, and immune systems. These actions are achieved by binding of estrogen receptors (ERs), including ERα, ERβ, and the G protein-coupled estrogen receptor (GPER) (Knowlton and Lee, 2012). In recent years, increasing evidence has suggested an intrinsic correlation between estrogen signaling and the LS. A large proportion of primary lymphatic diseases (e.g., Turner syndrome and lymphangioleiomyomatosis) are linked directly to estrogen synthesis (Shankar and Backeljauw, 2018). In addition, clinical research studies (Shrubb and Mason, 2006; Fontaine et al., 2020) have demonstrated that breast cancer survivors undergoing long-term hormone therapy with tamoxifen may be at an increased risk of lymphedema. Tamoxifen has been widely used in the treatment of estrogen receptor positive (ER+) breast cancer and is a well-known antiestrogen drug (Shagufta and Ahmad, 2018). Furthermore, it has been also reported that tamoxifen therapy might aggravate lymphedema, whereas E2 could improve LS dysfunction in animal models. To be specific, E2 could activate its receptor ERα and exert beneficial effects on LECs via genomic and non‐genomic pathways, including enhancing the expression levels of VEGFR3, LYVE-1, and VEGF-D and promoting the migration and branching of LECs by activating the AKT pathway. However, these aforementioned effects of E2 could be comprehensively suppressed by tamoxifen. (Morfoisse et al., 2018; Garmy-Susini, 2019). Therefore, estrogen signaling may be closely associated with the LS.

The treatment principle of LS damage is restoration of the structure and function of regional lymph nodes (LNs) as well as normal lymphatic flow. Treatments for lymphedema are, in general, palliative and include local compression therapy and physical therapy aimed at relieving symptoms but have limited efficacy (Grada and Phillips, 2017). Surgical treatments, such as debulking procedures and lymphovenous anastomosis, can be used against symptomatic lymphedema (Maeda et al., 2018; Schaverien and Coroneos, 2019).

With the development of microsurgery, lymph node transplantation (LNT) has become a promising approach. Healthy LNs are harvested from a remote area and transplanted to the affected extremity using microsurgical methods for revascularization (Hassani et al., 2020). Numerous animal and clinical studies have suggested that LNT can be employed to reconstruct local lymphatic structures and improve lymphatic functions (Honkonen et al., 2013; Dionyssiou et al., 2016; Najjar et al., 2018). The effects of estrogen or antiestrogen agents (e.g., tamoxifen) on structural reconstruction and functional recovery after LNT have not been investigated.

We explored if (i) estrogen contributes to the structural reconstruction and functional recovery of transplanted LNs and (ii) hormone therapy using tamoxifen inhibits the survival of transplanted LNs and affects the structural reconstruction of regionally transplanted LNs. We hope this study could provide new insights into LNT for lymphedema after cancer surgery, especially in breast cancer with adjuvant hormone therapy.

## Materials and Methods

### Study Design

The study protocol was approved by the Animal Ethics Committee of Shanghai Medical College of Fudan University (Shanghai, China). Animal experiments were conducted in accordance with the *Guide for the Care and Use of Laboratory Animals* (US National Institutes of Health, Bethesda, MD, United States). The mice model will be restricted to female mice because this article is focusing on the effect of estrogen and women breast cancer-associated hormone therapy. A total of thirty-six female C57BL/6 mice (4 weeks) were purchased from Shanghai Jihui Laboratory Animal Care (Shanghai, China). They were housed in a controlled environment. Food and water were made available freely.

The experiments were carried out under general anesthesia with 4% chloral hydrate (0.2 ml/20 g). Bilateral ovariectomy was performed on mice (Supplementary Figure S1A). Then, three groups of 16 were created: OVX + TMX (treated with tamoxifen), OVX + E2 (treated with 17β-estradiol), and control (treated with olive oil as a solvent). Administration doses were based on the study by Florent Morfoisse and colleagues (Morfoisse et al., 2018). Agents were administered *via* the subcutaneous route at 0.2 ml per time, twice a week for 6 weeks. Ovariectomy and transplantation of non-vascularized popliteal lymph nodes (PLNs) were performed after two weeks. After four weeks of surgery, mice were killed. Reconnection of transplanted PLNs and lymphatic vessels was identified, and tissues were collected for histology as well as immunofluorescence and immunohistochemical analyses.

### Ovariectomy

After the induction of general anesthesia, the fur on the back was shaved off. The skin was disinfected with chlorhexidine solution. A single midline dorsal incision (0.5 cm) was made using an operating scalpel. The incision was in the lower back, directly below the bottom of the rib cage. Then, the subcutaneous connective tissue from the underlying muscle on each side was freed using blunt forceps. The ovaries were located under the thin muscle layer. Next, a small (<0.5 cm) incision was made on each side to gain entry into the peritoneal cavity. The ovarian fat pad was removed carefully through the incision. Next, a single ligature was made around the oviduct to prevent bleeding after ovary removal. The ovary was removed by gently severing the oviduct using sterile micro-scissors. The uterus and the remaining part of the oviduct were placed back into the abdominal cavity, and the muscle layer was sutured. The steps stated above were repeated on the contralateral side, and the incision on the back was sutured. The mouse was placed on a heating pad until recovery (Supplementary Figures S1B–E).

### Transplantation of Non-Vascularized Popliteal Lymph Nodes

After the induction of general anesthesia, the fur on the popliteal fossa was shaved off, and ten microliters of 1% methylene blue was administered into the subcutaneous tissue of the left paw using a 1.0-ml syringe and 30-G needle. The PLN and lymphatic vessels were identified. After 4 min, using a sharp dissecting scalpel, an incision (3–4 mm) was made through the skin of the popliteal fossa. The stained PLN was found by separating regional fat and soft tissue. Furthermore, using micro-scissors, the afferent and efferent lymphatic vessels as well as peripheral blood vessels were excised and the PLN removed. Then, the removed PLN was placed back in its original position. These procedures were undertaken using a microsurgical technique under a microscope. The incised skin on the popliteal fossa was sutured with 5–0 nylon. The mouse was placed on a heating pad until recovery (Supplementary Figures S1F–I).

### Postoperative Assessment of Afferent and Efferent Lymphatic Reconnections

After four weeks of surgery, real-time staining of each transplanted PLN was observed. The fur in the popliteal fossa was shaved off. Ten microliters of 1% Evans Blue solution were injected into the subcutaneous tissue of the left paw of each mouse with a 1.0-ml syringe and a 30-G needle. The skin on the left hind limb was removed. The Evans Blue staining of each transplanted PLN and lymphatic vessels was observed. The transplanted PLNs were divided into those with and those without complete afferent and efferent lymphatic reconnections based on Evans Blue staining. The transplanted PLNs were collected with the surrounding soft tissue.

### Morphology of Transplanted Popliteal Lymph Nodes

PLNs were fixed with 4% paraformaldehyde and embedded in paraffin. Paraffin sections of a thickness of 4 μm were stained with hematoxylin and eosin (H&E). According to the scoring method used for transplanted LNs described by Tobbia D and colleagues (Tobbia et al., 2009), PLNs were scored and ranked on a scale of 0–3: 3 = normal appearance; 2 = some abnormality (evidence of ischemic damage and loss of cellularity); 1 = partial PLNs or severe damage (fibrosis); 0 = PLNs absent (resorbed into tissue). Based on the work of Tobbia D and colleagues (Tobbia et al., 2009), transplanted PLNs with a score of 2 or 3 were deemed to be “surviving PLNs,” and those with a score of 0 or 1 were deemed to be “non-surviving PLNs.” Besides, the size of each transplanted PLN in the largest cross-section was measured for mice in all groups, and the mean sizes in each group were calculated and compared.

### Immunofluorescence Analyses

Tissue sections underwent immunofluorescence staining for cluster of differentiation (CD) 45R (B cells), CD3 (T cells), and lymphatic vessel endothelial receptor (LYVE)-1(lymphatic endothelial cells). Sections were deparaffinized in xylene (three changes, 20-min each), and rehydrated in a graded series of ethanol solutions (100, 95, 80, and 70%, 5-min each). Antigens were retrieved using citric acid buffer (pH 6) and microwave heating. Before immunofluorescence analyses, sections were blocked with normal goat serum (catalog number: AR1009; Boster Biological Technology, Wuhan, China) for 30 min at room temperature.

The following primary antibodies were used (all at 1: 200 dilution): anti-human/mouse CD45R (B220) purified (clone: RA3-6B2; 07131-20-100; PeproTech, Rocky Hill, NJ, United States); anti-mouse CD3 purified (clone: 17A2; 05112-20-100; PeproTech) and mouse LYVE-1 monoclonal antibody (clone: 223322; MAB2125-SP; R&D Systems, Minneapolis, MN, United States) overnight at 4°C. Then, the sections were incubated (all at 1:100 dilution) with secondary fluorescent (CY3)-labeled sheep anti-mouse immunoglobulin (Ig)G (BA1031; Boster Biological Technology), fluorescent (CY3)-labeled sheep anti-rat IgG (Bs-0293G; BioSS, Edinburgh, Scotland), and fluorescent (fluorescein isothiocyanate)-labeled sheep anti-rat IgG (Bs-0293G; BioSS) for 1 h at 37°C. Then, 4′,6-diamidino-2-phenylindole (C1002; Beyotime Institute of Biotechnology, Beijing, China) was added to sections. Images were captured using a fluorescence microscope (BX53; Olympus, Tokyo, Japan).

### Immunohistochemical Staining

Sections underwent immunohistochemical staining for MECA-79 [high endothelial venules (HEVs)]. Sections were deparaffinized in xylene (three changes, 20-min each), and rehydrated in a graded series of ethanol solutions (100, 95, 80, and 70%, 5-min each). Antigens were retrieved using citric acid buffer (pH 6) and microwave heating. Primary antibodies and rat monoclonal MECA‐79 antibody (clone MECA‐79; 1:50 dilution; sc‐19602; Santa Cruz Biotechnology, Santa Cruz, CA, United States) were used overnight at 4°C. Sections were incubated with a biotinylated rabbit anti rat IgG secondary antibody for 30 min at room temperature, and then incubated with the ABC kit (SA1025; Boster Biological Technology) for 30 min at room temperature. Staining was achieved using a diaminobenzidine substrate kit (DA1010; Solarbio, Beijing, China).

### Immunofluorescence and Immunohistochemical Analyses

Digital images were analyzed by Image-Pro Plus 6.0 (Media Cybernetics, Rockville, ML, United States). According to previous studies (Lee et al., 2012; Ishikawa et al., 2019), “total HEVs” were defined as having an outline >100 μm^2^ and “dilated HEVs” were defined as having a lumen >80 μm^2^. The minimum luminal cross‐sectional area for functional HEVs was defined as 80 μm^2^. We counted the number of total HEVs and dilated HEVs for each PLN. Besides, the ratio of the number of dilated HEVs to the total number of HEVs was calculated. For immunofluorescent-stained sections, sections stained with LYVE-1 were scanned to select five “hotspots” (areas of the highest density) under a high-power microscope. The density was calculated as the mean number of lymphatic vessels in hotspots per field.

### Statistical Analyses

Statistical analyses were undertaken using STATA (College Station, TX, United States). Mouse survival in the three groups was compared using the Fisher’s exact test. The dry weight of the uterus, the mean size and mean number of HEVs, and the number of lymphatic vessels of transplanted PLNs in groups were compared by one-way analysis of variance (ANOVA). The ratio of dilated HEVs to total HEVs in groups was compared using the Kruskal–Wallis test. Multiple comparisons were made using Bonferroni’s correction and the Tukey test. *p* < 0.05 was considered significant.

## Results

### Ovariectomized Mice Treated With Estrogen or Tamoxifen

We wished to explore the effects of estrogen treatment and antiestrogen treatment on the structural reconstruction and functional restoration of transplanted PLNs. We created a bilateral ovariectomized model in mice, and administered tamoxifen, 17β-estradiol, or olive oil, respectively. After 6 consecutive weeks of treatment, mice were euthanized, and the condition of the uterus and fallopian tubes was observed to ascertain the efficacy of surgery and the reliability of our experimental model. A hypertrophic uterus and fallopian tubes were observed in the OVX + E2 group, as well as a significant increase in the dry weight of the uterus, which suggested the stimulatory effects of estrogen. The uterus and fallopian tubes of mice in the OVX group and OVX + TMX group showed obvious atrophy compared with those of mice in the control group. These results suggest that the experimental model and drug administration were efficacious and reliable (Supplementary Figures S1J–R).

### Reconnection of Transplanted PLNs and Lymphatic Vessels

**FIGURE 1:**
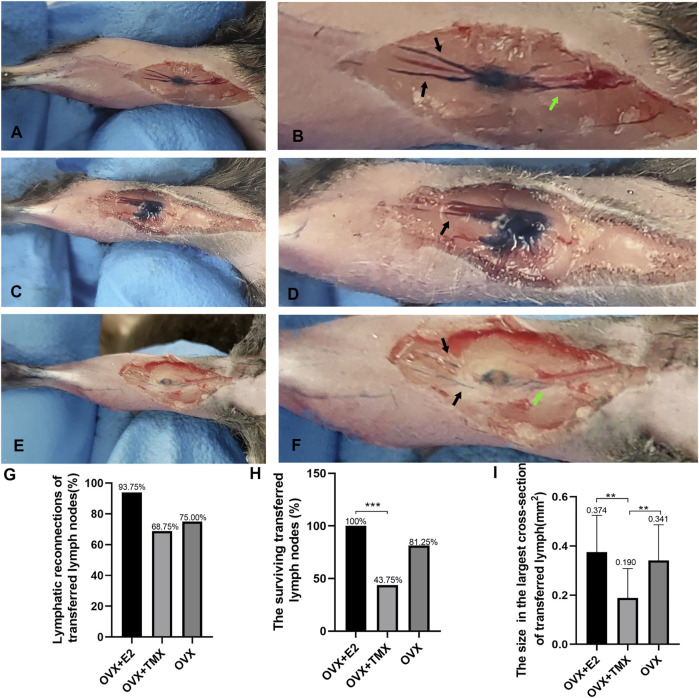
Validation of the experimental model with drug administration and the observation of the reconnections between lymphatic vessels and transferred lymph nodes. **(A,B)** Example of the reconnection between lymphatic vessels and lymph nodes. **(C,D)** Reconnections between lymph nodes with afferent and efferent lymphatics were not fully established and leakage exists. **(E,F)** Reconnection between lymphatic vessels and lymph nodes in the OVX + TMX group. Stained afferent lymphatic vessels (black arrows) and efferent lymphatic vessels (green arrows). **(G)** Quantification of lymphatic reconnections of three groups. **(H)** Quantification of surviving transferred lymph nodes of three groups. **(I)** Average sizes of transferred PLNs in each group in the largest cross sections. *p < 0.05, **p < 0.01, ***p < 0.001.

The completed reconnections between transplanted PLNs with afferent and efferent lymphatic vessels were the prerequisite for restoration of normal lymphatic drainage. To ascertain if estrogen treatment and antiestrogen treatment influenced the reconnections between PLNs with lymphatic vessels, afferent and efferent lymphatic vessels were observed on caudal and cephalic sides after injection of Evans Blue dye in mouse groups. Transplanted PLNs and lymphatic vessels would be dyed only if the completed connections had been reestablished (Figures 1A–D). In the OVX + E2 group, 15 of 16 regenerated transplants (93.75%) were connected to lymphatic vessels, whereas 11 of 16 (68.75%) mice in the OVX + TMX group had reconnections between PLNs with afferent and efferent lymphatic vessels (Figure 1G). In the control group, 12 of 16 (75%) transplanted PLNs achieved connections for lymphatic drainage to the leg. Interestingly, we observed that the staining condition of some transplanted LNs in group OVX + TMX was still worse compared with that in other groups, although both their afferent and efferent lymphatic vessels were stained using blue dye. Specifically, in some lymph nodes, only the marginal parts of lymph nodes were stained with blue dye, while the central parts were not dyed (Figures 1E, F).

### Morphology of Transplanted Popliteal Lymph Nodes

**FIGURE 2:**
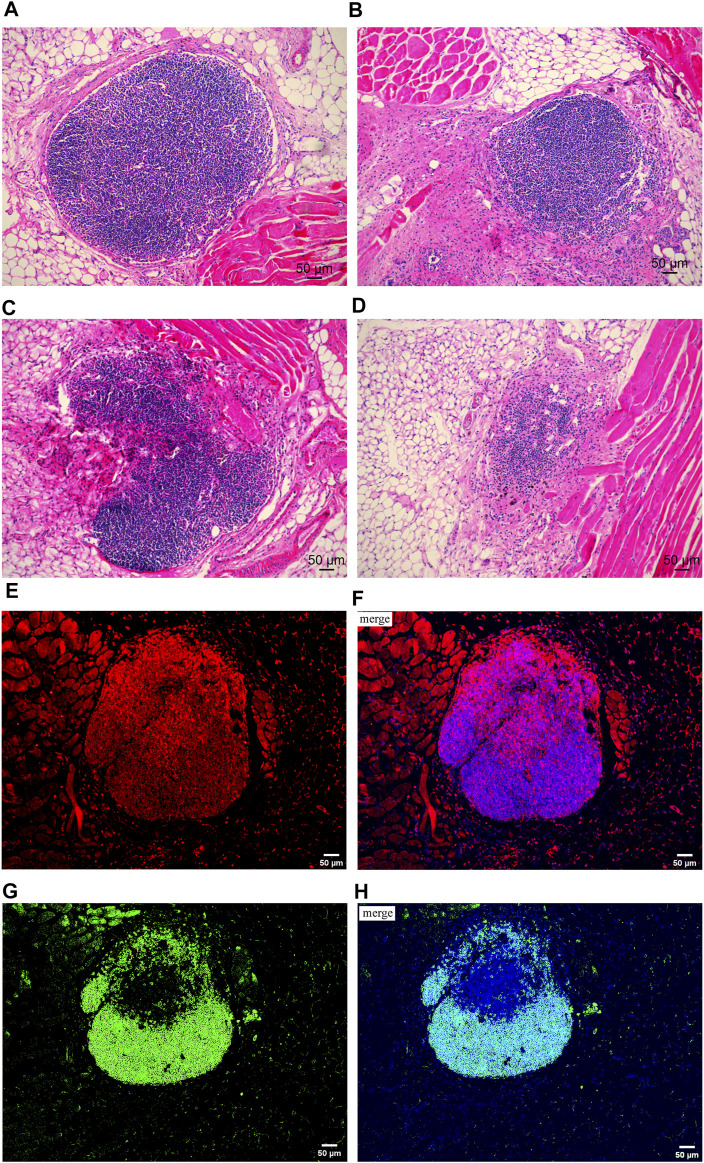
Hematoxylin and eosin staining on transplanted lymph nodes and immunofluorescence staining on T cells and B cells. **(A)** Example of normal node ranked 3. **(B)** Example of node ranked 2, nodes with some abnormality (evidence of ischemic damage and loss of cellularity). **(C)** Example of node ranked 1, partial nodes or nodes with severe damage. **(D)** Example of node ranked 0, lymph nodes absent (tissue resorbed). **(E,F)** Distribution of T cells in transplanted lymph nodes. **(E)** Red fluorescence represented CD3^+^ cells (T cells) and **(F)** was the merged image; the red fluorescence represented target protein CD3, and the blue fluorescence represented cell nucleus. **(G,H)** Distribution of B cells in transplanted lymph nodes. **(G)** Green fluorescence represented CD45 ^+^ cells (B cells) and **(H)** was the merged image; the green fluorescence represented target protein CD45, and the blue fluorescence represented cell nucleus.

A previous study revealed an obvious positive correlation between lymphatic transport function and LN health (Tobbia et al., 2009). According to the scoring method for transplanted LNs described by Tobbia D and colleagues, in the OVX + E2 group, all nodes were ranked 2 or 3 on histology, which indicated that the PLNs were, in general, healthy (Figures 2A,B). Transplanted PLNs in the OVX + TMX group fared poorly, with 4 of 16 ranked 0, 5 ranked 1, 5 ranked 2, and only 2 classified as 3 (Figures 2C, D). In the OVX group, 8 of 16 were rated 3, 5 of 16 were rated 2 2 of 16 were rated 1, and 1 of 16 were rated 0. With regard to PLN survival, 16/16 survived in the OVX + E2 group, 7/16 survived in the OVX + TMX group, and 13/16 survived in the OVX group. A significant difference in PLN survival was observed between the OVX + E2 group and the OVT + TMX group (*p* < 0.01, Fisher’s exact test, Bonferroni correction) (Figure 1H).

The mean maximum cross-sectional area (in mm^2^) of transplanted PLNs in the OVX + E2 group was 0.374 (0.21–0.67), in the control group was 0.341 (0.04–0.69), and that in the OXV + TMX group was 0.190 (0.00–0.41), which was significantly less than that in the other two groups (OVX + E2 *vs*. OVX + TMX, *p* < 0.01; OVX *vs*. OVX + TMX, *p* < 0.01; one-way ANOVA, Bonferroni correction) (Figure 1I). These results suggest that antiestrogen treatment might influence the survival of transplanted PLNs.

### Distribution of T Cells and B Cells in Transplanted Popliteal Lymph Nodes

We wished to discover if the transplanted PLNs had normal immune function. By immunofluorescence staining, we observed the distribution of T cells and B cells in transplanted PLNs. Follicle formation was observed in the cortex of transferred PLNs with afferent lymphatic reconnections but not in the cortex of PLNs without afferent lymphatic reconnections. In surviving transplanted PLNs, T cells were located mainly in the deep paracortex (T-cell zone), whereas B cells were observed mainly in the superficial cortex (B-cell zone). Besides, the histologic architectures of surviving transplanted PLNs were, in general, similar to those of normal PLNs (Groom, 2015) (Figures 2E–H).

### Number of HEVs and the Ratio of Dilated HEVs/Total HEVs in Transplanted Popliteal Lymph Nodes

**FIGURE 3:**
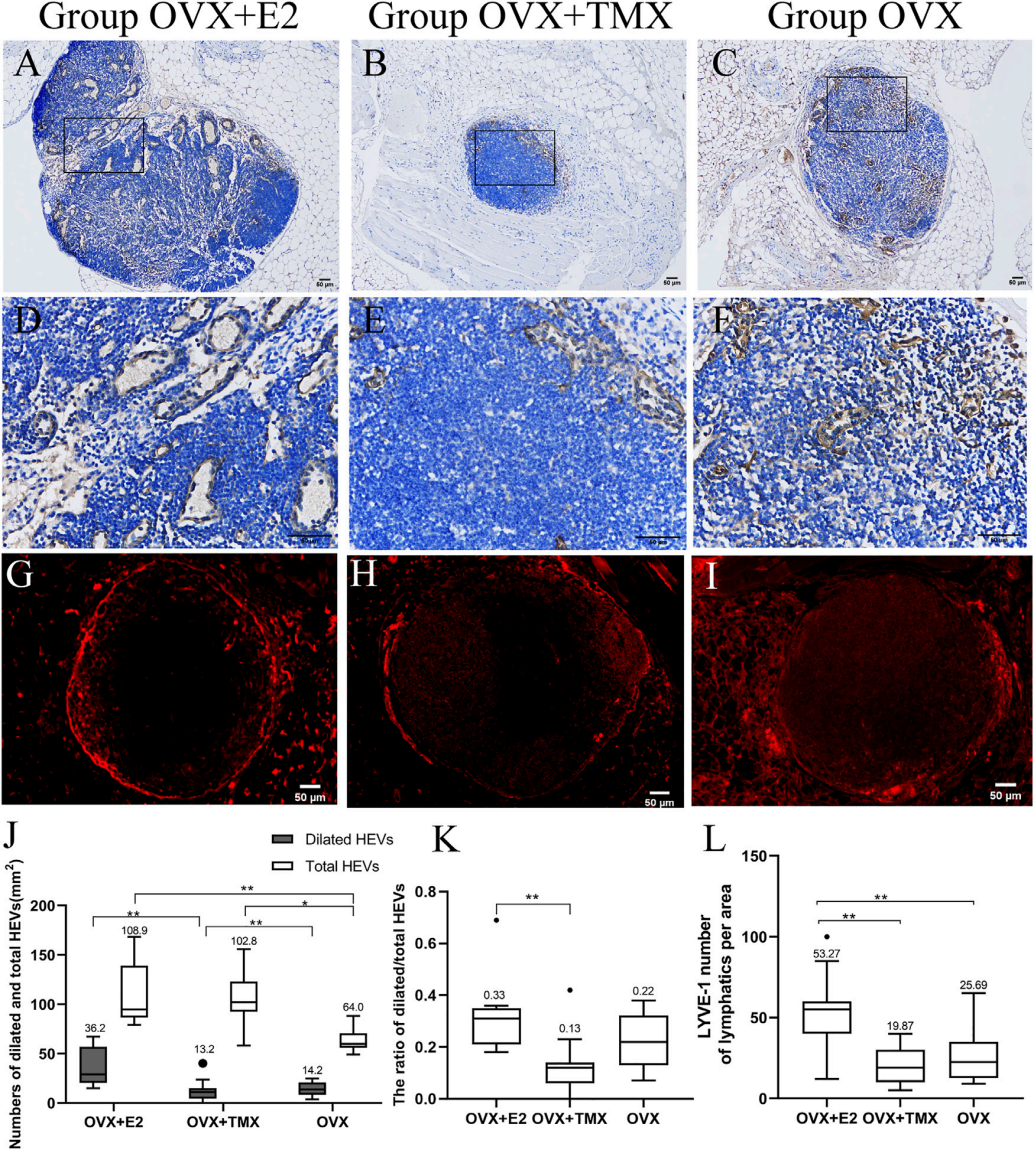
High endothelial venules (MECA-79+) and lymphatics (LYVE-1+) of transferred lymph nodes. **(A,D)** High endothelial venules of transferred lymph nodes in the OVX + E2 group. **(B,E)** High endothelial venules of transferred lymph nodes in the OVX + TMX group. **(C,F)** High endothelial venules of transferred lymph nodes in the OVX group. **(G)** Lymphatics of transferred lymph nodes in the OVX + E2 group. **(H)** Lymphatics of transferred lymph nodes in the OVX + TMX group. **(I)** Lymphatics of transferred lymph nodes in the OVX group. **(J)** Number of total and dilated high endothelial venules in three groups. **(K)** Ratio of dilated/total high endothelial venules in three groups. **(L)** Quantification of the lymphangiogenic response (LYVE-1+) in different groups. *p < 0.05, **p < 0.01, ***p < 0.001.

HEV diameter in the OVX + E2 group was obviously smaller than that in the other groups (Figures 3A–F). The number of dilated HEVs/mm^2^ in transplanted PLNs was 36.2 (14.9–67.1) in the OVX + E2 group, 13.2 (0–40.2) in the OVX + TMX group, and 14.2 (3.7–24.8) in the OVX group. Significant differences were seen in the OVX + E2 group compared with the OVX + TMX group (*p* < 0.01) and the OVX + TMX group compared with the OVX group (*p* < 0.01) using one-way ANOVA and the Bonferroni correction.

The number of total HEVs/mm^2^ in transplanted PLNs was 108.9 (79.2–168.3) in the OVX + E2 group, 102.8 (58.1–155.7) in the OVX + TMX group, and 64.1 (49.1–88.2) in the OVX group. Significant differences were observed between the OVX + E2 group compared with the OVX group (*p* < 0.01) and the OVX + TMX group compared with the OVX group (*p* < 0.01) using one-way ANOVA and the Bonferroni correction (Figure 3J).

The ratio of dilated HEVs/total HEVs in transplanted PLNs was 0.33 (0.18–0.69) in the OVX + E2 group, 0.13 (0–0.23) in the OVX + TMX group, and 0.22 (0.07–0.38) in the OVX group. The ratio of dilated HEVs/total HEVs in PLNs in the OVX + E2 group was significantly higher than that in the other groups. The lowest ratio of dilated HEVs/total HEVs was observed in the OVX + TMX group (OVX + E2 vs. OVX + TMX, *p* < 0.01, Kruskal–Wallis test, Bonferroni correction) (Figures 3D,K).

### Number of Lymphatic Vessels in Transplanted PLNs

LYVE-1 was present in the marginal area of transplanted PLNs (Figures 3G–I), a result that is consistent with the work of MAEDA T and colleagues (Maeda et al., 2018). The LYVE-1-positive area of lymphatic vessels in the OVX + E2 group was obviously greater than that in the other groups (Figures 3G–I). The mean number of lymphatic vessels per area in transplanted PLNs was 53.27 (12–100) in the OVX + E2 group, 19.87 (5–40) in the OVX + TMX group, and 25.69 (9–47) in the OVX group. A significant difference was noted between the OVX + E2 group compared with the OVX + TMX group (*p* < 0.001) and the OVX + E2 group compared with the OVX group (*p* < 0.001) using the one-way analysis of variance and the Tukey test. A comparison of the number of lymphatic vessels among the three groups is shown as Figure 3L.

## Discussion

LS damage is a common complication of cancer surgery, especially in breast cancer and melanoma, which can damage normal lymphatic backflow and lead to lymphedema. With advances in microsurgery, LNT has become a promising therapy for the reconstruction of local lymphatic structures and improvement of lymphatic functions. In recent years, increasing evidence has indicated an intrinsic correlation between estrogen signaling and the LS. Nevertheless, whether estrogen or antiestrogen agents can influence the structural reconstruction and functional restoration of transplanted LNs is not known. We constructed a model involving PLN transplantation after ovariectomy to explore this question.

The reconnections between transplanted PLNs with afferent and efferent lymphatic vessels are the prerequisite for restoration of normal lymphatic drainage. In the OVX + E2 group, 91.6% of transplanted PLNs reconnected with afferent and efferent lymphatic vessels, whereas the percentage was 68.75% in the OVX + TMX group and 71.42% in the OVX group. The prevalence of success of reconnections between transplanted LNs and lymphatic vessels has been reported to range from 22 to 100% (Rabson et al., 1982; Tammela et al., 2007; Maeda et al., 2018; Ishikawa et al., 2019). This discrepancy in data may be a result of the transplantation method, injury severity, and surgical proficiency. We undertook orthotropic LNT and preserved surrounding lymphatic vessels and soft tissues, which might have contributed to the relatively high number of reconnections.

We wished to further explore the effects of estrogen signaling on the survival of transplanted PLNs. Hence, we assessed the morphology and size of each transplanted PLN in the largest H&E‐stained cross section. We found that the percentage of surviving transplanted PLNs in the OVX + TMX group showed an obvious decrease compared with that in the other groups, accompanied by significant cell loss and LN size reduction. Therefore, tamoxifen might interfere with the survival and structural remodeling of transplanted PLNs. This action of tamoxifen might be achieved by directly affecting LECs. Garmy-Susini and colleagues (Morfoisse et al., 2018; Garmy-Susini, 2019) reported that tamoxifen could abrogate estradiol-induced beneficial effects on LECs by binding to its receptor ERα to block both genomic and non-genomic pathways. Specifically, tamoxifen could decrease expression levels of VEGFR3, LYVE-1, and VEGF-D and also alter lymphatic endothelial shape, in particular filopodia formation, to reduce the migration and branching of LECs by inhibiting the phosphorylation of the AKT pathway.

Furthermore, another possible mechanism is the toxic effects of TMX on adipose tissues. Increasing evidence indicates that there is an intrinsic correlation between adipose tissue and the lymphatic system. According to previous studies (Takeda et al., 2015; Li et al., 2020), adipose-derived stem cells (ADSCs) could promote proliferation, migration, and tube formation of LECs *in vitro* by secreting various lymphangiogenic factors. In addition, it has been demonstrated that ADSCs can increase the number of lymphatic vessels and promote the restoration of lymphatic drainage *in vivo* (Hayashida et al., 2017; Hu and Pan, 2020). Clinical research indicates that breast cancer survivors undergoing hormone therapy may be at an increased risk of poor fat graft survival during surgical reconstruction (Zhu et al., 2010). Besides, previous studies also revealed that TMX had cytotoxic effects on ASDCs *in vitro*, involving the promotion of apoptosis and inhibition of proliferation, as well as the suppression of the differentiating capacity of ASDCs (Pike et al., 2015). Additionally, it has also been demonstrated that TMX had a pro-death effect on adipocytes and induced acute weight loss in fat pads *in vivo* (Ye et al., 2015). Therefore, high doses of TMX administration might interfere with adipose tissues surrounding LNs, thereby inhibiting the survival of transplanted LNs indirectly. Nevertheless, further research is still needed to validate this potential mechanism.

Moreover, according to our results, the survival percentage of transplanted LNs is dramatically lower in group OVX + TMX, while the prevalence of success of reconnections between transplanted LNs and lymphatic vessels is not significantly less than that in group OVX + E2. A possible explanation is the inhibitory effect of tamoxifen on vascular endothelium. Specifically, the survival of transplanted LNs is closely related not only to the reconnections between lymphatics and LNs, but also to the restoration of the blood supply of transplanted LNs (Ishikawa et al., 2019). Previous studies have indicated that tamoxifen could suppress the proliferation and migration of vascular endothelial cells *in vitro* and reduce the levels of VEGF in serum *in vivo* (Mcnamara et al., 2001). And tamoxifen-treated animals also exhibited a significantly reduced rate of micro-vessel growth in response to VEGF (Blackwell et al., 2000). Therefore, tamoxifen might interfere with the restoration of the blood supply of transferred LNs, thereby influencing the survival of transplanted LNs.

HEVs form a branching network of post-capillary venules that is fully integrated into the normal vascular blood bed of LNs (Girard et al., 2012). Communications between lymph vessels and veins are achieved by HEVs, which can pump lymph into the blood circulation (Jiang et al., 2021). Besides, HEVs are sites of large-scale migration of various lymphocytes from the blood into LNs (Ager and May, 2015; Milutinovic et al., 2021). Therefore, HEV regeneration is crucial for functional restoration of transplanted PLNs. We assessed the number of total HEVs and functional HEVs per unit area in transplanted PLNs of different groups. And our results supported that E2 treatment might promote the regeneration and maintenance of functional HEVs of transplanted PLNs, whereas tamoxifen could inhibit those effects. The protective effects of estrogen on the blood vessel system have been demonstrated in numerous studies and involve mechanisms such as anti-inflammation and antioxidant stress (Miller and Duckles, 2008; Knowlton and Lee, 2012). Studies have also indicated that the number of HEVs in LNs in female mice is higher than that in male mice (Kittas and Henry, 1979). Estrogen has been shown to promote proliferation and tube formation of vascular endothelial cells (Zhou et al., 2017), which might explain the effects of estrogen/tamoxifen on HEVs of transplanted PLNs in our study.

Lymphangiogenesis is a crucial link in the functional restoration of transplanted PLNs. New lymphatic vessels are generated from lymphatic endothelial progenitors or preexisting lymphatic vessels. This action facilitates reconnections of lymphatic vessels between transplanted PLNs and the transplantation region (Tammela and Alitalo, 2010; Aschen et al., 2014). In the present study, significantly more lymphatic vessels of transplanted PLNs were present in the OVX + E2 group than those in other groups, which suggested that E2 might contribute to lymphangiogenesis. Generally consistent with our results, Garmy-Susini and colleagues also revealed that E2 could promote the branching and migration of LECs and enhance expression of vascular endothelial growth factor-D, vascular endothelial growth factor receptor 3, and LYVE-1, all of which have a crucial role in lymphangiogenesis (Morfoisse et al., 2018).

Collectively, our data suggest that: (i) tamoxifen treatment might lead to cellular loss of transplanted LNs and interfere with the structural reconstruction and functional restoration of transplanted PLNs, thereby inhibiting the survival of LNs; (ii) estrogen treatment facilitates the maintenance and regeneration of functional HEVs as well as lymphangiogenesis. These discoveries might provide new insights into LNT for lymphedema after cancer surgery, especially in breast cancer. Hormone therapy might be a potential risk factor affecting the survival and functional restoration of transplanted LNs. A combination of some adjunctive measures promoting the survival of transplanted LNs may be needed in patients undergoing hormone therapy to achieve a better therapeutic effect.

Our study had three main limitations. First, the model we created was not a lymphedema model. Therefore, whether the effects of hormone therapy on transplanted PLNs would influence the therapeutic effect of LNT on lymphedema must be validated. Second, we evaluated the transplanted PLNs at a single time-point, that is, 4 weeks after surgery; a longer period may be necessary to observe the regeneration of transplanted PLNs. Third, our conclusions were based on a mouse model of LNT and were not a clinical study.

## Conclusion

Using the ovariectomized LNT model, we demonstrated that tamoxifen treatment might lead to cellular loss of transplanted LNs and interfere with the structural reconstruction and functional restoration, thereby inhibiting the survival of transferred LNs. Estrogen treatment facilitated the maintenance and regeneration of functional HEVs as well as lymphangiogenesis. These discoveries might provide new insights into LNT for lymphedema after cancer surgery, especially in breast cancer with hormone therapy.

## Data Availability

The raw data supporting the conclusion of this article will be made available by the authors, without undue reservation.

## Supplementary Material

Supplementary Figure S1 Construction of the ovariectomized mice model of the non-vascularized popliteal lymph node transplantation. **(A)** Schematic representation of the experimental procedures. **(B)** Incision was made in the lower back. **(C)** Single ligature was performed around the oviduct. **(D)** Ovary removed by gently severing the oviduct by using sterile small scissors. **(E)** Incision in the back skin closed after repeating the aforementioned steps on the contralateral side. **(F)** 3–4 mm incision was made through the skin of the popliteal fossa using a sharp dissecting scalpel. **(G)** Stained PLN was found by separating regional fat and soft tissue. **(H)** Afferent and efferent lymphatics as well as peripheral blood vessels were cut off, and the PLN was removed. **(I)** Incised skin was sutured after the removed PLN was placed back into its original position. **(J–M)** Uterine and fallopian tubes *in vitro* from OVX + E2, OVX + TMX, and OVX groups and normal mice, respectively. **(N–Q)** Uterine and fallopian tubes *in vivo* from OVX + E2, OVX + TMX, and OVX groups and normal mice, respectively. **(R)**. Dry weight of the uterus in different groups and normal mice. **p* < 0.05, ***p* < 0.01, ****p* < 0.001.
