# Supplementary figures and images for: Hormone Therapy: A Potential Risk Factor Affecting Survival and Functional Restoration of Transplanted Lymph Nodes
Source: Front Pharmacol. 2022 Mar 31;13:853859. doi: 10.3389/fphar.2022.853859 (PMC9008310; doi:10.3389/fphar.2022.853859)

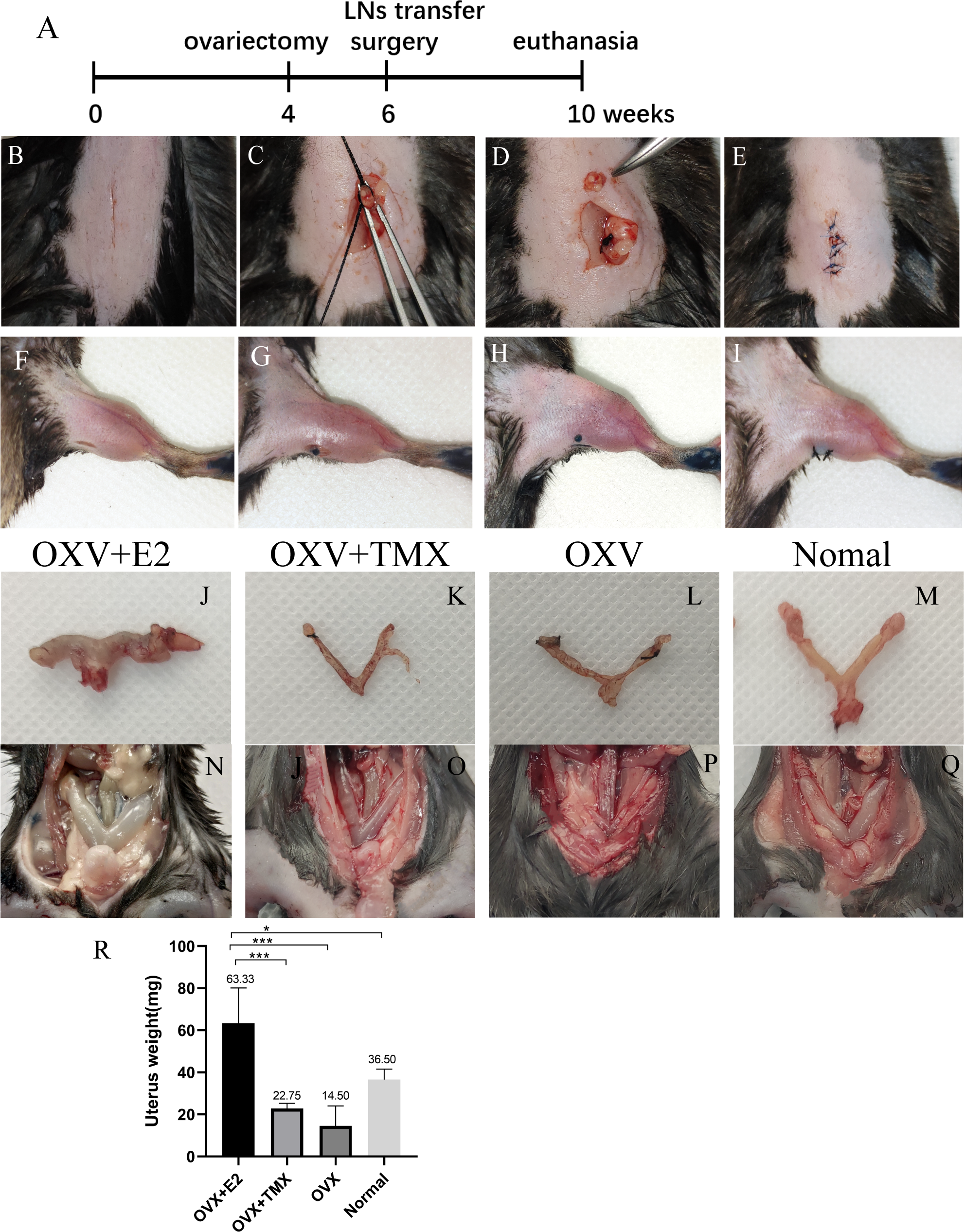

Supplement: Supplementary file 1 [file Image1.TIF]
